# Cesarean birth in the Global Network for Women’s and Children’s Health Research: trends in utilization, risk factors, and subgroups with high cesarean birth rates

**DOI:** 10.1186/s12978-020-01021-7

**Published:** 2020-12-17

**Authors:** Margo S. Harrison, Ana L. Garces, Shivaprasad S. Goudar, Sarah Saleem, Janet L. Moore, Fabian Esamai, Archana B. Patel, Elwyn Chomba, Carl L. Bose, Edward A. Liechty, Nancy F. Krebs, Richard J. Derman, Patricia L. Hibberd, Waldemar A. Carlo, Antoinette Tshefu, Marion Koso-Thomas, Elizabeth M. McClure, Robert L. Goldenberg

**Affiliations:** 1 University of Colorado School of Medicine, Denver, CO USA; 2 Instituto de Nutrición de Centroamérica y Panamá, Guatemala, Guatemala; 3 KLE Academy Higher Education and Research J N Medical College, Belagavi, Karnataka India; 4 Aga Khan University, Karachi, Pakistan; 5 RTI International, Durham, NC USA; 6 Moi University School of Medicine, Eldoret, Kenya; 7 Lata Medical Research Foundation, Nagpur, India; 8 University Teaching Hospital, Lusaka, Zambia; 9 University of North Carolina at Chapel Hill, Chapel Hill, NC USA; 10 Indiana School of Medicine, University of Indiana, Indianapolis, IN USA; 11 Thomas Jefferson University, Philadelphia, USA; 12 Boston University School of Public Health, Boston, MA USA; 13 University of Alabama at Birmingham, Birmingham, AL USA; 14 Kinshasa School of Public Health, Kinshasa, Democratic Republic of the Congo; 15 Eunice Kennedy Shriver National Institute of Child Health and Human Development, Bethesda, MD USA; 16 Department of Obstetrics and Gynecology, Columbia University School of Medicine, New York, NY USA; 17 University of Colorado, Mail Stop B198-2, Academic Office 1, 12631 E. 17th Avenue, Rm 4211, Aurora, CO 80045 USA

**Keywords:** Cesarean birth, Low- and middle-income countries, Trends, Risk factors, Vaginal birth after cesarean, Robson classification

## Abstract

**Background:**

The objectives of this analysis were to document trends in and risk factors associated with the cesarean birth rate in low- and middle-income country sites participating in the Global Network for Women’s and Children’s Health Research (Global Network).

**Methods:**

This is a secondary analysis of a prospective, population-based study of home and facility births conducted in the Global Network sites.

**Results:**

Cesarean birth rates increased uniformly across all sites between 2010 and 2018. Across all sites in multivariable analyses, women younger than age twenty had a reduced risk of cesarean birth (RR 0.9 [0.9, 0.9]) and women over 35 had an increased risk of cesarean birth (RR 1.1 [1.1, 1.1]) compared to women aged 20 to 35. Compared to women with a parity of three or more, less parous women had an increased risk of cesarean (RR 1.2 or greater [1.2, 1.4]). Four or more antenatal visits (RR 1.2 [1.2, 1.3]), multiple pregnancy (RR 1.3 [1.3, 1.4]), abnormal progress in labor (RR 1.1 [1.0, 1.1]), antepartum hemorrhage (RR 2.3 [2.0, 2.7]), and hypertensive disease (RR 1.6 [1.5, 1.7]) were all associated with an increased risk of cesarean birth, p < 0.001. For multiparous women with a history of prior cesarean birth, rates of vaginal birth after cesarean were about 20% in the Latin American and Southeast Asian sites and about 84% at the sub-Saharan African sites. In the African sites, proportions of cesarean birth in the study were highest among women without a prior cesarean and a single, cephalic, term pregnancy. In the non-African sites, groups with the greatest proportion of cesarean births were nulliparous women with a single, cephalic, term pregnancy and all multiparous women with at least one previous uterine scar with a term, cephalic pregnancy.

**Conclusion:**

Cesarean birth rates continue to rise within the Global Network. The proportions of cesarean birth are higher among women with no history of cesarean birth in the African sites and among women with primary elective cesarean, primary cesarean after induction, and repeat cesarean in the non-African sites.

## Background

Globally, cesarean birth rates are on the rise [1]. Many countries have exceeded the World Health Organization (WHO) recommended cesarean birth rate of 10–15%, with some regions experiencing rates over 40% [1, 2]. Conversely, very low resource regions that may have poor access to facility birth, and subsequently cesarean birth, often fall below the recommended range [3]. Cesarean birth rates within the Global Network for Women’s and Children’s Health Research (Global Network) have been increasing, paralleling the global trend [4]. The Global Network prospectively collects population-based data on home and facility births in six low- and middle-income countries that span Latin America, sub-Saharan Africa, and Southeast Asia in an ongoing registry [5]. Data from this Maternal Newborn Health Registry (MNHR) within the Global Network was previously analyzed to show that over a relatively short period of time (2010–2016), cesarean birth rates doubled at all non-sub-Saharan African sites, almost reaching 30% in one Indian site [4]. Rates at the sub-Saharan African sites were well below 5%, despite also nearly doubling across the time period studied [4]. Given these trends, this study serves to update the analysis of cesarean birth rates in the Global Network, to observe risk factors associated with cesarean birth (our primary outcome), and to consider subgroups contributing to the cesarean birth rates (our secondary outcome).

## Methods

### Study overview

This analysis was conducted using data from a prospective study conducted in communities at seven sites in six low- and middle-income countries for births from January 2010 through December 2018 (North and South Ubangi Province, the Democratic Republic of the Congo (DRC); Chimaltenango, Guatemala; Nagpur, and Belagavi, India; western Kenya; Thatta District, Pakistan; and sites near Lusaka, Zambia), through the Global Network. The DRC site initiated enrollment in 2014, and data were collected on the Robson criteria in all sites starting in 2017. The Robson criteria classify women by common obstetric variables into ten mutually exclusive groups [2, 6].

### Setting

The Global Network’s prospective registry, the MNHR, includes pregnancy related data and outcomes from rural or semi-urban geographical areas. Each site includes between 6 and 24 distinct communities [5]. Each community generally represents the catchment area of a primary healthcare center, and about 300–500 annual births [5]. The objective of the MNHR is to enroll pregnant women as early as possible during the pregnancy and to obtain data on pregnancy outcomes for all deliveries of registered women, regardless of birth location (i.e., home, health clinic, or hospital) [5].

### Population/recruitment

The analyses presented here used MNHR data to determine trends in cesarean birth across study sites over time, risk factors associated with cesarean birth among registrants, and the prevalence of cesarean birth among the Robson subgroups since 2017. The population studied included women screened for the MNHR who were eligible, consented, and delivered in the study period. Data were excluded from women who were enrolled but lost to follow-up prior to delivery, maternal deaths prior to labor and delivery, miscarriages, medically terminated pregnancies, and those with missing data for delivery mode.

### Outcomes

The primary outcome of this analysis was cesarean birth as the mode of delivery, including rates over time, and risk factors associated with cesarean birth. The secondary outcomes were rates of vaginal birth after previous cesarean birth and the proportion of cesarean birth in each Robson subgroup.

### Analysis

We used descriptive statistics to produce counts and percentages of cesarean births per Global Network site per year using standard contingency table techniques. Then we observed independent variables associated with cesarean birth, and performed comparisons of sociodemographic and antenatal covariates that we hypothesized might be associated with mode of delivery. Relative risks, 95% confidence intervals and p values were obtained from log binomial models as a function of each individual risk factor using generalized estimating equations to account for the correlation of outcomes within cluster. Backward selection was used to identify risk factors to include in the final multivariate model starting with all risk factors that were found to be associated with cesarean birth (p < 0.05 from the individual risk factor log binomial models). We performed a separate model for African (DRC, Kenya and Zambia) and non-African (Nagpur and Belagavi, India, Pakistan and Guatemala) sites given the substantial differences in cesarean rates in the African sites compared to the other sites. The final multivariable Poisson models used to evaluate the relationship of associated or potential risk factors with cesarean birth included non-colinear, statistically significant covariates and used generalized estimating equations to account for the correlation of outcomes within cluster. We then observed the vaginal birth after cesarean birth rate among women with a history of prior cesarean birth.

To consider subgroups contributing to cesarean birth rates, our secondary outcome, we utilized the WHO-recommended Robson Classification System, which is a method of comparing cesarean birth rates over time within and across institutions to classify women by common obstetric variables into ten mutually exclusive groups [2, 6]. These variables include parity, history of prior cesarean birth, onset of labor, number of fetuses, gestational age, and fetal presentation [2, 6]. Cesarean birth rates among the classification subgroups can help identify populations contributing to the overall cesarean birth rate [2, 6]. In an effort to better understand increased utilization of cesarean birth across the Global Network sites, we applied the Robson Classification System to our pregnancy cohort. All data analyses were done with SAS software v.9.4. (SAS Institute, Cary, NC, USA).

### Ethics

The appropriate institutional review boards/ethics research committees of the participating institutions approved the MNHR study. Individual informed consent for study participation was requested and obtained from each study participant. A Data Monitoring Committee, appointed by the National Institute of Child Health and Human Development reviewed the study semi-annually [5].

## Results

**Fig. 1:**
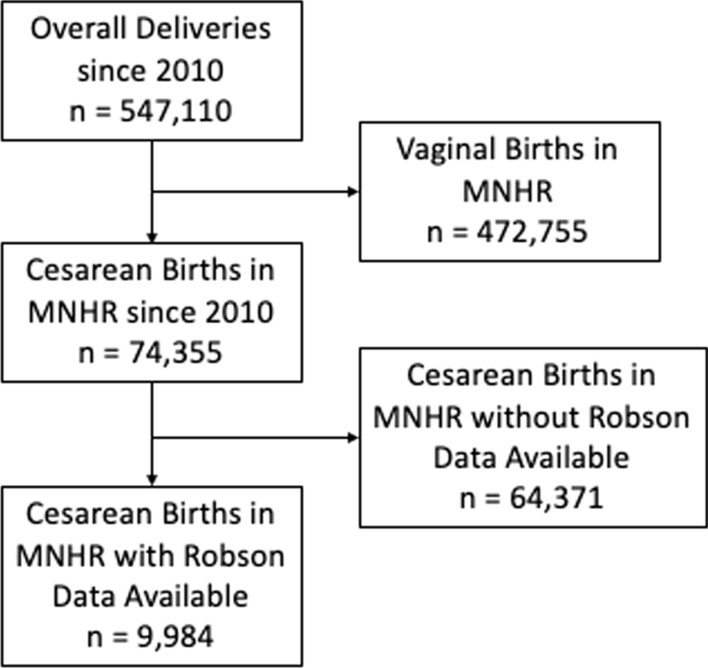
CONSORT diagram

Figure 1 is the enrollment diagram for the population included in this analysis. Of 547,110 births that have been documented in the MNHR since 2010, 74,355 (13.6%) women gave birth by cesarean. Of those with a cesarean, 9,984 (13.4%) gave birth since 2017 and included the data necessary to classify them into the Robson groups.

**Fig. 2:**
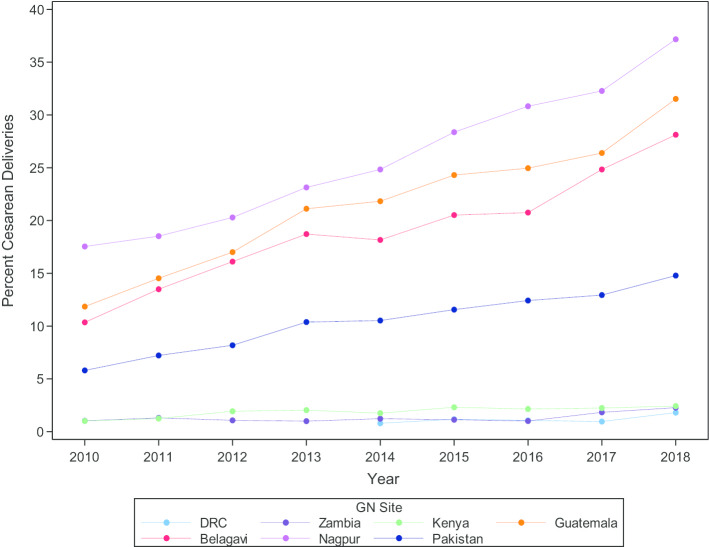
Trend in cesarean birth rate within the global network, by site, ongoing clusters, 2010–2018

Figure 2 shows cesarean birth rates over time, which indicates a continued rising trend since previously assessed [4]. Rates ranged from 1.8% in the DRC to 37.2% of all births in Nagpur, India, in 2018, compared to the 2010 rate of 17.5% in Nagpur and 0.8% in the DRC in 2014. The Indian and Guatemalan sites had the highest rates, all greater than 28% in 2018. The cesarean birth rate in Pakistan was almost 15% in 2018, and the sub-Saharan African sites had the lowest rates, all below 2.5%.

Table 1 shows the results of generalized linear models used to evaluate the relationship of associated or potential risk factors with cesarean birth, which was the first step in assessing our primary outcome. Separate models were run for all sites, African sites, and non-African sites. The overall results show that all covariates except preterm birth (RR 1.0 [1.0, 1.1]) were significantly associated with cesarean birth in unadjusted comparisons. Maternal age over 35, as compared to age 20–35, was associated with a reduced risk of cesarean birth (RR 0.9 [0.86, 0.96], p < 0.001). Any education was associated with increased risk of cesarean birth as compared to no formal schooling; women with primary/secondary schooling had over a two-fold increased risk (RR 2.1 [1.9,2.3], p < 0.001) and women with university or higher-level education had almost a four-fold increased risk (RR 3.9 [3.5, 4.4], p < 0.001). Any parity below three was associated with increased risk of cesarean delivery than women with parity of 3 or more; in nulliparous women the relative risk was 2.4 [2.2, 2.5] and in women with parity of 1–2, the relative risk was 1.7 [1.6, 1.8], p < 0.001. Women with four or more antenatal visits had a higher risk of cesarean than women with less visits (RR 1.9 [1.7, 2.1], p < 0.001). Multiple pregnancy was also associated with an elevated risk of cesarean birth (RR 2.0 [1.9, 2.2], p < 0.001). Abnormal progress in labor, defined as occurrence of obstructed labor, prolonged labor or failure to progress reported versus none reported, (OL/PL/FTP) was strongly associated with cesarean delivery (RR 5.9 5.0, 6.9) as was abnormal lie (RR 5.0 [4.4, 5.5]), antepartum hemorrhage (RR 1.9 [1.7, 2.0]), and hypertensive disease [any of hypertension, preeclampsia, or eclampsia versus none reported (HTN/PEC/EC: RR 3.0 [2.7, 3.4]), p < 0.001. In the separate models run by region, all relative risks were consistent in terms of increased risk for cesarean birth except maternal age. In the African sites, advanced maternal age was associated with an increased risk of cesarean birth (RR 1.4 [1.2, 1.7], p < 0.001) and in the non-African sites the risk was reduced (RR 0.9 [0.8, 0.9], p < 0.001). Gestational age was not associated with the outcome across all study sites.

**Table 1:** Risk factors associated with cesarean birth, 2010–2018

	Overall			African sites			Non-African sites		
	Cesarean/total deliveries (%)	RR (95% CI)^a^	p value^a^	Cesarean/total deliveries (%)	RR (95% CI)^a^	p value^a^	Cesarean/total deliveries (%)	RR (95% CI)^a^	p value^a^
Maternal age (years)	0.001			< 0.001			< 0.001		
< 20	10.0%	1.0 (1.0, 1.0)	0.7	1.5%	1.0 (0.9, 1.1)	0.4	20.1%	1.0 (1.0, 1.0)	0.7
20–35	14.3%	Ref		1.5%	Ref		18.9%	Ref	
> 35	10.6%	0.9 (0.9, 1.0)	< 0.001	2.0%	1.4 (1.2, 1.7)	< 0.001	17.2%	0.9 (0.8, 0.9)	< 0.001
Maternal education	< 0.001			< 0.001			< 0.001		
No formal schooling	7.6%	Ref		0.9%	Ref		8.8%	Ref	
Primary/secondary	13.8%	2.1 (1.9, 2.3)	< 0.001	1.5%	1.5 (1.3, 1.7)	< 0.001	21.4%	2.0 (1.9, 2.2)	< 0.001
University+	32.2%	3.9 (3.5, 4.4)	< 0.001	4.8%	4.7 (3.4, 6.3)	< 0.001	37.1%	3.5 (3.2, 3.9)	< 0.001
Parity	< 0.001			< 0.001			< 0.001		
0	19.6%	2.4 (2.2, 2.5)	< 0.001	2.1%	1.7 (1.5, 1.9)	< 0.001	25.7%	2.7 (2.5, 2.8)	< 0.001
1–2	13.8%	1.7 (1.6, 1.8)	< 0.001	1.4%	1.1 (1.0, 1.2)	0.2	18.4%	2.0 (1.9, 2.1)	< 0.001
3+	5.5%	Ref	< 0.001	1.2%	Ref		8.7%	Ref	< 0.001
Antenatal visits	< 0.001			< 0.001			< 0.001		
< 4	7.7%	Ref	< 0.001	1.1%	Ref		12.0%	Ref	< 0.001
4+	20.1%	1.9 (1.7, 2.1)	< 0.001	2.2%	1.9 (1.7, 2.1)	< 0.001	26.5%	1.9 (1.7, 2.1)	< 0.001
Multiple pregnancy	< 0.001			< 0.001			< 0.001		
Yes	24.5%	2.0 (1.9, 2.2)	< 0.001	7.6%	5.2 (4.4, 6.2)	< 0.001	35.5%	1.9 (1.8, 2.1)	< 0.001
No	13.5%	Ref		1.5%	Ref		18.8%	Ref	
Abnormal lie	< 0.001			< 0.001			< 0.001		
Yes	64.0%	5.0 (4.4, 5.5)	< 0.001	29.2%	24.5 (19.7, 30.5)	< 0.001	70.5%	4.1 (3.8, 4.5)	< 0.001
No	12.5%	Ref		1.2%	Ref		17.6%	Ref	
OL/PL/FLP^b^	< 0.001			< 0.001			< 0.001		
Yes	56.1%	5.9 (5.0, 6.9)	< 0.001	21.3%	47.5 (35.2, 64.3)	< 0.001	63.9%	5.0 (4.3, 5.8)	< 0.001
No	9.8%	Ref		0.5%	Ref		14.1%	Ref	
Antepartum hemorrhage	< 0.001			< 0.001			< 0.001		
Yes	21.3%	1.9 (1.7, 2.0)	< 0.001	11.1%	7.8 (6.4, 9.5)	< 0.001	25.4%	1.7 (1.5, 1.8)	< 0.001
No	13.5%	Ref		1.4%	Ref		18.9%	Ref	
HTN/PEC/EC^c^	< 0.001			< 0.001			< 0.001		
Yes	42.5%	3.0 (2.7, 3.4)	< 0.001	13.6%	9.4 (7.3, 12.1)	< 0.001	45.7%	2.6 (2.4, 2.9)	< 0.001
No	12.8%	Ref		1.4%	Ref		18.0%	Ref	
Preterm	0.9			0.7			1.0		
Yes	12.6%	1.0 (1.0, 1.1)	0.9	1.6%	1.0 (0.9, 1.2)	0.7	17.7%	1.0 (1.0, 1.1)	1.0
No	13.9%	Ref		1.5%	Ref		19.3%	Ref	

^a^Relative risks and p values are obtained from log Binomial models as a function of each individual risk factor using generalized estimating equations to account for the correlation of outcomes within cluster

^b^OL/PL/FTP represents obstructed labor, prolonged labor, failure to progress

^c^HTN/PEC/EC represents hypertension, pre-eclampsia, eclampsia

Table 2 shows the results of the multivariable models that analyzed risk factors associated with cesarean birth in adjusted comparisons, the second step in evaluating our primary outcome. The only variable excluded was preterm delivery (p > 0.7). OL/PL/FTP was correlated with abnormal lie, likely because many abnormal lie pregnancies result in OL/PL/FTP [7]. Therefore, abnormal lie was excluded from the model because it is more specific than OL/PL/FTP, which can result from multiple etiologies. Overall, compared to the reference group, age less than 20 years was associated with a reduced risk (RR 0.9 [0.9, 0.9) and age greater than 35 was associated with an increased risk (RR 1.1 [1.1, 1.1]) of cesarean birth, p < 0.001. Similar to the results of Table 3, as years of education increased, so did the risk of cesarean birth; the most educated women had a 50% increased risk of cesarean birth compared to women with no formal schooling, across all sites (RR 1.5 [1.4, 1.7], p < 0.001). Similarly, results related to parity were unchanged in the multivariable model as compared to the unadjusted models (RR 1.2 [1.2, 1.3], p < 0.001). Finally, having greater than four antenatal visits (RR 1.2 [1.2, 1.3]), a multiple gestation (RR 1.3 [1.3, 1.4]), experiencing dysfunctional labor (RR 1.1 [1.03, 1.1]), having antepartum hemorrhage (RR 2.3 [2.0, 2.7]), or having hypertensive disease (RR 1.6 [1.5, 1.7]) increased the risk of cesarean birth, p < 0.001.

**Table 2:** Risk factors associated with cesarean birth, multivariable models

	Overall		African sites^b^		Non-African sites	
	RR (95% CI)^a^	p value^a^	RR (95% CI)^a^	p value^a^	RR (95% CI)^a^	p value^a^
Maternal age (years)		< 0.001		< 0.001		< 0.001
< 20	0.9 (0.9, 0.9)	< 0.001	0.97 (0.96, 0.99)	< 0.001	0.9 (0.9, 0.9)	< 0.001
20–35	Ref		Ref		Ref	
> 35	1.1 (1.1, 1.1)	< 0.001	1.0 (1.0, 1.1)	< 0.001	1.1 (1.1, 1.2)	< 0.001
Maternal education		< 0.001		< 0.001		< 0.001
No formal schooling	Ref		Ref		Ref	
Primary/secondary	1.3 (1.2, 1.3)	< 0.001	1.0 (1.0, 1.0)	0.04	1.3 (1.2, 1.3)	< 0.001
University+	1.5 (1.4, 1.7)	< 0.001	1.1 (1.1, 1.2)	< 0.001	1.5 (1.4, 1.7)	< 0.001
Parity		< 0.001		0.002		< 0.001
0	1.3 (1.2, 1.4)	< 0.001	1.0 (1.0, 1.1)	< 0.001	1.4 (1.3, 1.5)	< 0.001
1–2	1.2 (1.2, 1.3)	< 0.001	1.0 (1.0, 1.0)	< 0.001	1.3 (1.2, 1.4)	< 0.001
3+	Ref		Ref		Ref	
4 + antenatal visits	1.2 (1.2, 1.3)	< 0.001	1.0 (1.0, 1.1)	< 0.001	1.3 (1.2, 1.3)	< 0.001
Multiple pregnancy	1.3 (1.3, 1.4)	< 0.001	1.2 (1.1, 1.3)	< 0.001	1.4 (1.3, 1.4)	< 0.001
OL/PL/FLP	1.1 (1.0, 1.1)	< 0.001	1.1 (1.0, 1.1)	0.01	1.1 (1.1, 1.1)	< 0.001
Antepartum hemorrhage	2.3 (2.0, 2.7)	< 0.001	2.2 (1.7, 2.8)	< 0.001	2.1 (1.9, 2.4)	< 0.001
HTN/PEC/EC	1.6 (1.5, 1.7)	< 0.001	1.2 (1.1, 1.3)	< 0.001	1.5 (1.4, 1.6)	< 0.001

^a^Relative risks and p values are obtained from log binomial models as a function of each individual risk factor using generalized estimating equations to account for the correlation of outcomes within cluster

^b^OL/PL/FTP represents obstructed labor, prolonged labor, failure to progress

^c^HTN/PEC/EC represents hypertension, pre-eclampsia, eclampsia

**Table 3:** Robson classification system for cesarean birth

Robson group	Description
1	Nulliparous women with single cephalic pregnancy, ≥ 37 weeks gestation in spontaneous labor
2	Nulliparous women with single cephalic pregnancy, ≥ 37 weeks gestation who either had labor induced or were delivered by cesarean section before labor
3	Multiparous women without a previous uterine scar, with single cephalic pregnancy, ≥ 37 weeks gestation in spontaneous labor
4	Multiparous women without a previous uterine scar, with single cephalic pregnancy, ≥ 37 weeks gestation who either had labor induced or were delivered by cesarean section before labor
5	All multiparous women with at least one previous uterine scar, with single cephalic pregnancy, ≥ 37 weeks gestation
6	All nulliparous women with a single breech pregnancy
7	All multiparous women with a single breech pregnancy, including women with previous uterine scars
8	All women with multiple pregnancies, including women with previous uterine scars
9	All women with a single pregnancy with a transverse or oblique lie, including women with previous uterine scars
10	All women with a single cephalic pregnancy < 37 weeks gestation, including women with previous scars

**Fig. 3:**
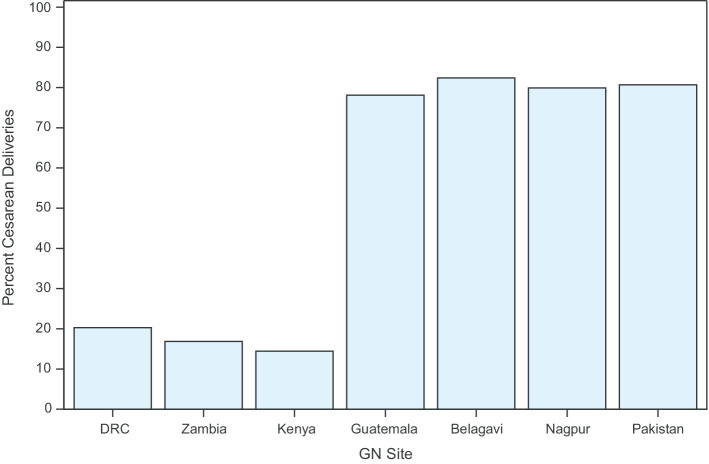
Rates of cesarean birth for multiparous women with a prior cesarean birth within the global network by site, 2017–2018

Figure 3 illustrates rates of vaginal birth after cesarean among women with a prior cesarean birth. The proportions of vaginal birth after cesarean ranged around 20% in the Latin American and south Asian sites and around 84% at the sub-Saharan African sites.

We applied the Robson Classification System for cesarean birth to determine the percentage of cesareans at each site in each group as shown in Table 4. The African sites had a greater proportion of cesarean births in groups one (nulliparous, single cephalic term pregnancy in spontaneous labor) and three (multiparous without a previous uterine scar, single cephalic term pregnancy in spontaneous labor), while the non-African sites had the highest rates in groups one, two (nulliparous, single cephalic term pregnancy with labor induced or cesarean before labor), and five (multiparous with a previous uterine scar, single cephalic term pregnancy). All sites seemed to have a relatively high proportion of cesarean birth in group ten (single cephalic preterm pregnancy) with Pakistan having the highest proportion of cesarean birth in this subgroup.

**Table 4:** Robson classification of cesarean birth within the global network, by site, 2017–2018: the percent of cesareans within each group

	Overall	African sites			Non-African sites			
		DRC	Zambia	Kenya	Guatemala	Belagavi	Nagpur	Pakistan
Robson group, N (%)	9981	135	161	198	4197	2368	2550	372
1. Nulliparous, single cephalic term pregnancy in spontaneous labor	22.0%	28.1%	28.6%	34.8%	15.7%	28.9%	26.2%	9.1%
2. Nulliparous, single cephalic term pregnancy with labor induced or cesarean before labor	20.6%	0.7%	1.2%	5.6%	23.5%	17.9%	22.7%	14.2%
3. Multiparous without a previous uterine scar, single cephalic term pregnancy in spontaneous labor	6.7%	29.6%	30.4%	23.2%	4.9%	8.1%	4.9%	3.2%
4. Multiparous without a previous uterine scar, single cephalic term pregnancy with labor induced or cesarean before labor	8.2%	1.5%	5.6%	3.0%	9.1%	8.4%	7.1%	11.6%
5. Multiparous with a previous uterine scar, single cephalic term pregnancy	23.2%	7.4%	8.1%	10.1%	25.7%	20.1%	24.7%	22.8%
6. Nulliparous with a single breech pregnancy	2.5%	0.7%	6.2%	2.0%	2.5%	3.0%	2.2%	1.6%
7. Multiparous with a single breech pregnancy	2.0%	4.4%	6.8%	5.6%	2.7%	1.2%	0.7%	3.0%
8. Multiple pregnancy	1.6%	8.9%	3.7%	5.6%	1.3%	1.8%	0.9%	1.6%
9. Single pregnancy with a transverse or oblique lie	2.6%	5.9%	1.2%	3.0%	3.8%	1.5%	1.1%	4.6%
10. Single cephalic preterm pregnancy	10.6%	12.6%	8.1%	7.1%	10.7%	9.1%	9.4%	28.2%

## Discussion

Cesarean birth rates within the Global Network appear to continue to rise across all sites, although rates are substantially lower in the African sites. For our primary outcome, the strongest predictors of cesarean birth in adjusted analyses in the African sites were multiple pregnancy, a university or higher level of education, antepartum hemorrhage, and hypertension in pregnancy. In the non-African sites, the factors most strongly associated with cesarean birth in adjusted analyses were advanced maternal age, a primary school or higher level of education, parity less than three, greater than four antenatal visits, multiple gestation, abnormal progress in labor, antepartum hemorrhage, and hypertensive disease.

For our secondary outcome, in all sites with cesarean birth rates at or above the historically recommended WHO rate of 10–15% (Pakistan, India, Guatemala), vaginal birth after cesarean rates were around 20%. Conversely, in the African sites where cesarean birth rates are very low, vaginal birth after cesarean rates were around 84%. We also found that primary or first cesarean (among nulliparous and multiparous women) accounted for the largest proportion of cesareans performed in the African sites, while pre-labor primary cesarean or primary cesarean after induction of labor among nulliparous women, and repeat cesarean among multiparous women accounted for a higher proportion of cesareans in the non-African sites. Overall, the proportion of cesarean births accounted for by preterm singletons was about 11%.

We used statistical modeling to identify risk factors associated with cesarean birth in the sub-Saharan African sites that might be of interest in considering interventions to modify cesarean birth rates, either to increase or decrease the rate given the site. For hypertensive disease in pregnancy, mode of delivery should be determined by routine obstetric considerations—hypertensive disease in itself is not an indication for cesarean birth [8–12]. However, the decision to proceed with cesarean birth in the setting of hypertensive disease must consider individualized risks and benefits of expediting birth. More research on common management protocols in the context of hypertensive disease at the study sites would be of interest. Considering antepartum hemorrhage, bleeding can result from a number of etiologies [13]. If it results in fetal death, vaginal birth is the recommended mode of delivery, as it is if there is not associated maternal and/or fetal compromise from the blood loss [13]. However, if the fetus and/or mother are determined to be at risk, the plan should be for immediate delivery, which may require urgent cesarean birth or assisted vaginal birth [13]. Regarding multiple pregnancy, a randomized trial of uncomplicated diamniotic twin pregnancies at greater than 32 weeks’ gestation with a cephalic presenting fetus did not find an increased risk of neonatal morbidity and mortality with vaginal as compared to cesarean birth [14]. Women with multiple gestations who qualify and have an appropriately trained provider may be candidates for vaginal birth [14]. Therefore, further research on how cases of hypertensive disease, antepartum hemorrhage, and multiple gestation are being managed in the sub-Saharan African sites would be of interest. There may be areas for further research on decision support tools or other interventions to reduce cesarean births under appropriate circumstances.

In the model involving the non-African sites, additional risk factors that might be modified include age, adherence to antenatal care, and management of abnormal progress in labor. Advanced maternal age is a known risk factor for cesarean birth, although this may be a proxy measure of other unknown confounders [15]. Many interventions exist for delaying the age of onset of child-bearing, but interventions to reduce the age of childbearing were not easily identified [16]. Interestingly, despite higher rates of preterm birth among older mothers in low- and middle-income countries, advanced maternal age was also associated with less stunting, better school progression, and higher adult height attainment [16]. This is a complex area with equipoise to support additional research and guidelines specific to low-resource settings related to age, but no clear recommendation or intervention can be suggested at this time, which suggests that this is an area rich for future research. Regarding antenatal care and labor management, the WHO has issued guidelines on these topics that offer recommendations on how to ensure that women have access to the right care at the right time in pregnancy and during labor and delivery [17–20]. How to ensure implementation of these evidence-based recommended is an area needing further research.

Performance of cesarean birth is always a complicated decision given the many factors contributing to any one woman’s labor and delivery, but mode of delivery after a history of cesarean birth is even more complex [21, 22]. The data on trial of labor after cesarean versus elective repeat cesarean birth is scarce in low- and middle-income country settings [21]. No clear international guidelines have been proposed, and guidelines from high-income countries are not prescriptive [21, 23–27]. Our data suggest that as the cesarean birth rate exceeds the recommended rate, repeat cesarean birth also becomes more common, accounting for the vast majority of deliveries that occur in women with a history of prior cesarean birth. Conversely, in communities where cesarean birth is underutilized, vaginal birth after cesarean may be the more common method of birth as compared to repeat cesarean. Mode of delivery after cesarean in low-resource settings represents an area where further research and guidance is needed.

Our final analysis was to use the WHO-recommended Robson Cesarean Birth Classification System to subset our cohort into ten mutually exclusive groups to see which women accounted for the greatest proportion of cesarean births in our Global Network sites. Other large studies have used the Robson classification, previously [28]. A WHO analysis of 21 countries to assess cesarean birth trends using the Robson Classification System found that Robson groups one (nulliparous, single cephalic term pregnancy in spontaneous labor), three (multiparous without a previous uterine scar, single cephalic term pregnancy in spontaneous labor), and five (multiparous with a previous uterine scar, single cephalic term pregnancy) had the highest absolute contribution to the overall cesarean birth rate in the low human development index countries [28]. We found that in our African sites, there were a high proportion of primary cesarean births, and in our non-African sites, there was a high proportion of repeat cesarean births, pre-labor (elective) primary cesarean births, and primary cesarean births occurring among women who were induced. Therefore, our results are similar to those of the WHO analysis [28]. The WHO authors concluded that repeat cesarean birth is an increasingly important determinant of cesarean birth in moderate or low human development index countries, and strategies should be implemented to reduce medically unnecessary primary cesarean birth [28]. They also suggested that improved case selection for induction and pre-labor cesarean birth could reduce cesarean birth rates, which are excellent gaps for future research [28]. We feel these conclusions apply to our analysis as well, although repeat cesarean birth was a less relevant contributor to cesarean birth rates in our African sites.

Although this analysis represents a large dataset and a globally representative sample, it is limited by the short timeframe during which the Robson classification variables were collected, the multiple comparisons made, the lack of information available on maternal preference for elective cesarean birth, and the lack of context in which to interpret these trends and findings. For example, determinants of rising rates are likely very different in Latin America as compared to southeast Asia, but we have only basic sociodemographic, antepartum, intrapartum, and postpartum data with which to analyze our outcomes. Similarly, with respect to cesarean birth, optimizing cesarean birth utilization in the African sites based on prior research requires greater use of cesarean birth, while appropriate use of cesarean birth in our non-African sites may necessitate reduced use of the procedure. We also note that our a priori stratification of sites may have resulted in missing potential associations and conclusions, and variability of site data included in these grouping may not be fully explored. Having limited contextual data on the determinants of these trends constrains our ability to draw specific conclusions or make evidence-based recommendations from this analysis.

However, this analysis provides excellent preliminary data for further research. It highlights the gaps in knowledge about determinants of cesarean birth rates in varied low- and middle-income country settings and identifies areas for future research. These include the fields of prevention of the primary cesarean, appropriate use of vaginal birth after cesarean, and identifying subpopulations ideal for induction of labor and pre-labor cesarean specific to the low- and middle-income country context that account for variability in cesarean access and utilization across regions. It also offers some direction on additional risk factors that can be targets for interventions or for guideline development in low-resource settings that include mode of delivery in the setting of multiple gestation, hypertensive disease, and antepartum hemorrhage.

## Conclusion

In conclusion, cesarean birth rates appear to be increasing within the Global Network sites. Advanced maternal age, education, parity less than three, greater than four antenatal visits, multiple gestation, antepartum hemorrhage, and hypertensive disease in pregnancy are associated with cesarean birth in our study population. Proportions of cesarean birth are higher among women with no history of cesarean birth in the African sites and among women with primary elective cesarean, primary cesarean after induction, and repeat cesarean in the non-African sites.

## Data Availability

Components of the dataset are publicly available through the NICHD DASH website and can obtained with proper ethics approval and data use agreements.

## Abbreviations

DRC: Democratic Republic of Congo
WHO: World Health Organization
MNHR: Maternal Newborn Health Registry
